# *In silico* identification of novel antimicrobial peptides from the venom gland transcriptome of the spider *Argiope bruennichi* (Scopoli, 1772)

**DOI:** 10.3389/fmicb.2023.1249175

**Published:** 2023-07-28

**Authors:** Min Kyoung Shin, In-Wook Hwang, Bo-Young Jang, Kyung-Bin Bu, Jung Sun Yoo, Jung-Suk Sung

**Affiliations:** ^1^Department of Life Science, Dongguk University-Seoul, Goyang, Republic of Korea; ^2^Species Diversity Research Division, National Institute of Biological Resources, Incheon, Republic of Korea

**Keywords:** antimicrobial peptide, *Argiope bruennichi*, spider venom gland transcriptome, *in silico* analysis, Aranetoxin-Ab2a, Aranetoxin-Ab3a, multidrug-resistant *Pseudomonas aeruginosa*

## Abstract

As the emergence and prevalence of antibiotic-resistant strains have resulted in a global crisis, there is an urgent need for new antimicrobial agents. Antimicrobial peptides (AMPs) exhibit inhibitory activity against a wide spectrum of pathogens and can be utilized as an alternative to conventional antibiotics. In this study, two novel AMPs were identified from the venom transcriptome of the spider *Argiope bruennichi* (Scopoli, 1772) using *in silico* methods, and their antimicrobial activity was experimentally validated. Aranetoxin-Ab2a (AATX-Ab2a) and Aranetoxin-Ab3a (AATX-Ab3a) were identified by homology analysis and were predicted to have high levels of antimicrobial activity based on *in silico* analysis. Both peptides were found to have antibacterial effect against Gram-positive and -negative strains, and, in particular, showed significant inhibitory activity against multidrug-resistant *Pseudomonas aeruginosa* isolates. In addition, AATX-Ab2a and AATX-Ab3a inhibited animal and vegetable fungal strains, while showing low toxicity to normal human cells. The antimicrobial activity of the peptides was attributed to the increased permeability of microbial membranes. The study described the discovery of novel antibiotic candidates, AATX-Ab2a and AATX-Ab3a, using the spider venom gland transcriptome, and validated an *in silico*-based method for identifying functional substances from biological resources.

## 1. Introduction

Animal venom is used for defense and predation and is widely utilized among animal phyla, including spiders, snakes, cone snails, and scorpions (Utkin, 2015). It is a cocktail of biological components with specific physiological activities, most of which are toxins that exhibit inherent selectivity (Lewis and Garcia, 2003; Saez et al., 2010). Studying venom toxins not only helps us understand the biological function of each component but also offers a platform to discover useful derivatives. To take advantage of the underlying mechanisms, toxins have been applied in various fields, from molecular probes to pharmaceutical applications (Wang and Wang, 2016). For instance, a peptide analog from the venom of the pit viper *Bothrops jararaca* was discovered to inhibit the angiotensin-converting enzyme and was developed into a drug, captopril, for treating heart failure and hypertension (Escoubas and King, 2009).

Antimicrobial peptides (AMPs) are bioactive molecules that participate in the early defense against pathogens and are universally found in living organisms (Ghosh, 2020). AMPs are generally short, amphipathic peptides that are diverse in sequence and structure (Fjell et al., 2012; Shin et al., 2020). This diversity gives rise to distinct interactions with molecular targets, such as membranes and proteins of microorganisms. A well-established mechanism of AMPs is to increase the membrane permeability of bacteria by attaching to negatively charged lipid layers and causing structural changes (Sato and Feix, 2006). In this sense, AMPs are often called host defense peptides (HDPs) and actively participate in innate immunity (Sitaram and Nagaraj, 2002; Bahar and Ren, 2013).

There is great importance in discovering new antibiotic reagents as the emergence of multidrug-resistant (MDR) bacteria has become a global health threat. However, pharmaceutical companies are stepping out of investments to develop new antibiotics owing to low profits. Under such circumstances, AMPs are a promising candidate for next-generation antibiotics owing to their rapid and efficient elimination of pathogens (Mwangi et al., 2019). Therefore, it is crucial to identify novel AMPs and elucidate their function as well as the mechanism of action.

Acquisition of large amounts of biological resources and data has become feasible with the advancement of technology, aiding AMP discovery. In addition to traditional proteomic analysis, which directly investigates the composition of a biological sample, the analysis of transcriptome data allows the screening of functional candidates in a high-throughput manner. *In silico* analysis provides information on the structural and physicochemical properties of peptides encoded by transcripts (Gupta et al., 2021; Lee et al., 2021). For example, homology analysis using the Basic Local Alignment Search Tool (BLAST) allows functional prediction based on sequence similarities with known sequences. Furthermore, functional prediction and characterization of protein sequences have become possible through implementing artificial intelligence technologies such as machine learning, promoting extended utilization of biological resources (Vamathevan et al., 2019; Schmidt and Hildebrandt, 2021).

In this study, the venom gland transcriptome of the spider *Argiope bruennichi* (Scopoli, 1772) was subjected to *in silico* analyses for identifying potent AMPs. Aranetoxin-Ab2a (AATX-Ab2a) and Aranetoxin-Ab3a (AATX-Ab3a) were selected based on the results of homology analysis and structural, functional prediction. The peptides significantly inhibited the growth of bacterial and fungal strains, including MDR bacteria. AATX-Ab2a and AATX-Ab3a exhibited antimicrobial activity by increasing the membrane permeability of pathogens while exhibiting low cytotoxicity to human cells. Our study demonstrated the identification of novel AMPs with strong antimicrobial activity from the transcriptome of the spider venom gland by applying *in silico*-based methods.

## 2. Materials and methods

### 2.1. *In silico* analysis of peptides

For homology and structural analysis, the following programs and tools were utilized: Protein calculator,^1^ XtalPred,^2^ SignalP 4.1,^3^ SpiderP,^4^ Pepfold,^5^ and HeliQuest.^6^ For prediction of AMPs, the following programs and tools were utilized: database of antimicrobial peptides (ADAM),^7^ AmpGram,^8^ the support vector machine in CAMPR3,^9^ and the database of antimicrobial activity and structure of peptides (DBAASP).^10^

### 2.2. Peptide synthesis and preparation

The peptides used in the study were synthesized by Biostem (Ansan, Korea) using the solid phase method. The peptides were synthesized with purity >95% and were verified by high-performance liquid chromatography and mass spectroscopy. Both peptides were reconstituted in distilled water to a final concentration of 1 mM, and the aliquots were stored at −80°C before further experiments.

### 2.3. Microbial strains and cell lines

The bacterial and fungal strains used in this study were obtained from the American Type Culture Collection (ATCC), the Korean Culture Center of Microorganisms (KCCM), or the Culture Collection of Antimicrobial Resistant Microbes (CCARM). *Escherichia coli* KCCM 11234, *Pseudomonas aeruginosa* ATCC 9027, *Bacillus cereus* KCCM 21366, *Staphylococcus aureus* KCCM 11335, and MDR *P. aeruginosa* isolates CCARM 2007 and 2095 were grown in tryptic soy agar (TSA, Difco Laboratories, Detroit, MI, United States) at 37°*C. candida albicans* ATCC 10231 was grown in yeast extract peptone dextrose broth (YPDB, Difco Laboratories) at 37°C. *Fusarium oxysporum* isolate KCCM 60555 was cultured on potato dextrose agar (PDA, Difco Laboratories) plates at 25°C for 5 days and collected using a cell scraper. HaCaT (CLS 300493, Eppelheim, Germany) was purchased from the Cell Lines Service (CLS) and cultured in Dulbecco’s modified Eagle medium supplemented with 10% fetal bovine serum (Gibco, Grand Island, NY, United States) supplemented with 1% penicillin and streptomycin (Gibco). Adipose-derived mesenchymal stem cell (hADMSC) was obtained from CEFO Co. (Seoul, Korea) and was maintained in CEFOgro™ Human MSC Growth Medium (CEFO Co.). Both cell lines were cultured at 37°C under humidified air with 5% CO_2_.

### 2.4. Microbial activity assay

The colony-forming unit (CFU) assays were used to determine the antimicrobial activity of peptides. Cultures of mid-log phase bacterial and fungal cells were diluted to 2 × 10^6^ CFU/mL and 2 × 10^4^ cells/mL, respectively, and were mixed with an equal volume of peptides. After incubating for 3 h, the samples were spread onto agar plates and cultured overnight. The relative colony formation was calculated based on the number of counted colonies on the control and sample plates.

### 2.5. Measurement of bacterial membrane permeability

Outer membrane permeability of Gram-negative bacteria was assessed by 1-N-phenyl-naphtylamine (NPN; Sigma-Aldrich, St. Louis, MO, United States) uptake assay. NPN buffer was diluted in 5 mM HEPES buffer (Sigma-Aldrich) to reach a final concentration of 10 μM. Bacteria culture was washed with HEPES buffer and was diluted to reach 1 × 10^8^ CFU/mL. For each well, 100 μL of bacteria suspension was transferred, followed by the addition of 50 μL of NPN and peptides. The fluorescence intensity was measured for 15 min at 460 nm with excitation at 355 nm using Infinite F200 Pro multimode microplate reader (Tecan, Switzerland).

For cytoplasmic membrane permeabilization assay, 3,3’-Dipropylthiadicarbocyanine iodide (DiSC_3_(5); Sigma-Aldrich) dye was used. Bacterial culture was diluted in HEPES buffer containing 0.4 μM DiSC_3_(5) and 0.1 mM EDTA to a final concentration of 1 × 10^7^ CFU/mL. Bacterial cells of 100 μL were transferred into a 96-well plate and incubated for 30 min at 37°C. The fluorescence was measured immediately after an equal volume of peptide was added at excitation wavelength of 622 nm and emission wavelength of 670 nm.

### 2.6. PI uptake and staining assay

To determine membrane integrity after peptide treatment, fungal cells were harvested and mixed with peptides at the desired concentration. Melittin, a well-known AMP in bee venom, was used as a positive control. After incubating for 3 h, cells were washed with PBS and were treated with 50 μg/mL PI for 30 min at 4°C. Samples were washed three times with PBS to remove unbound dye from the cells. In case of *C. albicans*, PI uptake was measured by BD LSRFortessaTM Flow Cytometer (BD Biosciences, San Jose, CA, United States). For PI staining of *F. oxysporum*, 10 μL of the each sample was observed with a C1Si confocal microscope (Nikon, Japan).

### 2.7. Cell viability test

To evaluate the cytotoxic effects of the peptides, hADMSC and HaCaT cells were seeded in 96-well plates at a density of 1 × 10^5^ cells/mL. Different concentrations of the peptides were treated to the cells were incubated for 24 h. Quanti-Max WST-8 Cell Viability assay solution (Biomax, Seoul, Korea) was added to each well, followed by incubation for 1 h in cell incubator. Absorbance was determined at 450 nm using a microplate reader (Molecular Devices), and the relative cell viability was calculated based on the control.

### 2.8. Statistical analysis

All experiments were conducted in triplicate and the results are expressed as mean ± standard error of the mean (SEM). The statistical significance of the data was evaluated by performing a one-way ANOVA test followed by Tukey’s post-test using GraphPad Prism 9.3.1 (GraphPad Software, La Jolla, CA, United States). *p*-values of <0.05 were considered statistically significant.

## 3. Results

### 3.1. *In silico* identification of potential AMPs from the *Argiope bruennichi* venom gland transcriptome

**Figure 1:**
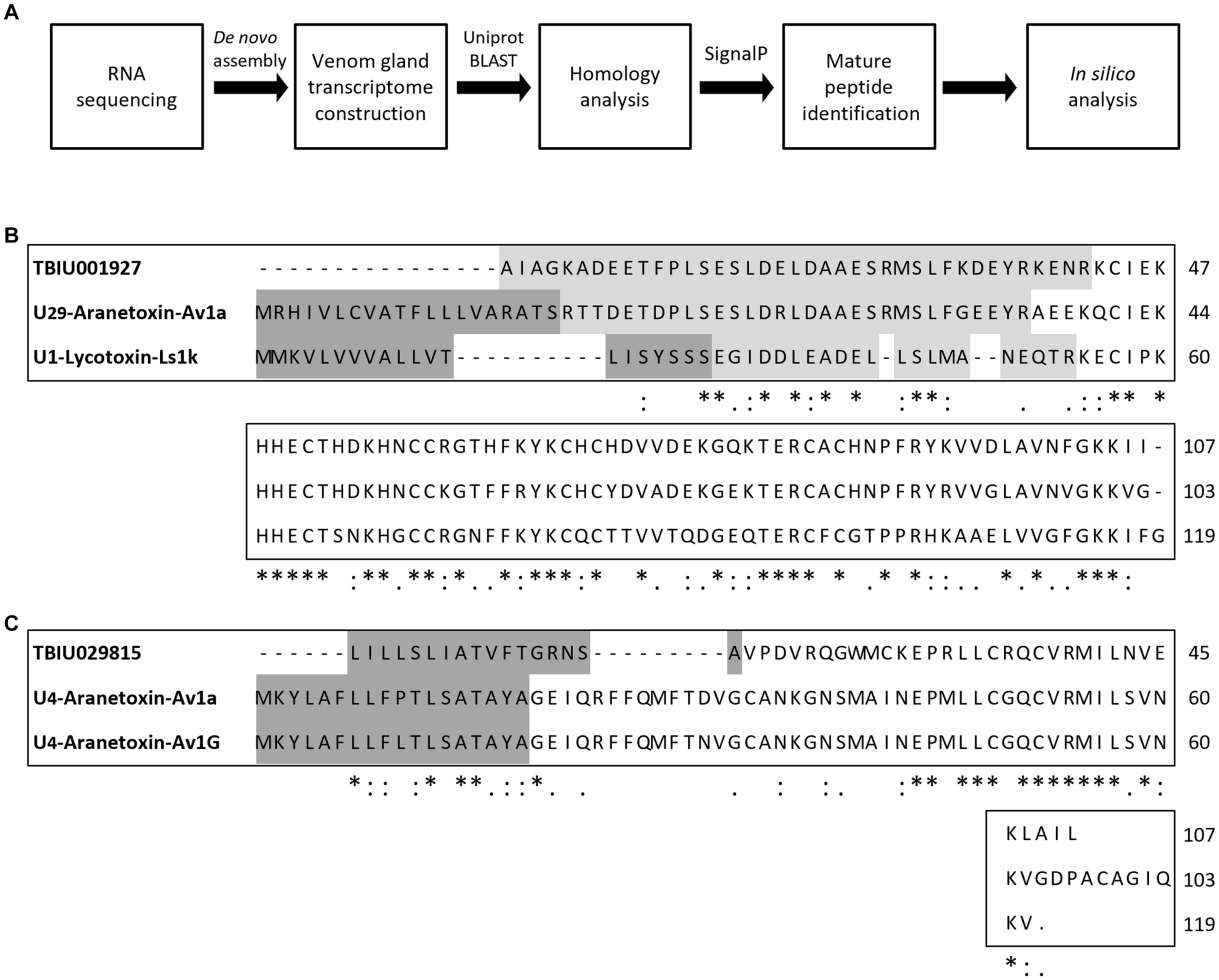
Study workflow and multiple sequence alignment of predicted peptides from *Argiope bruennichi* venom gland transcripts. **(A)** Scheme of the study design. After RNA sequencing and *de novo* assembly, *A. bruennichi* venom gland transcripts were searched against the Uniprot database. Putative peptide sequences that showed homology with known spider-derived toxin peptides were selected. Mature peptide sequences were identified and subjected to *in silico* analysis for characterizing physiochemical properties as well as predicting functionality. **(B)** TBIU001927 and **(C)** TBIU029815 were shown to encode peptides with significant homology with known spider toxin peptides among venom gland transcripts. The encoded mature peptide region of both transcripts was identified by analyzing the signal and/or propeptide regions of putative sequences. *, :, . each indicates perfect alignment, strong similarity, and weak similarity among sequences.

In order to identify novel AMPs, the *A. bruennichi* transcriptome obtained in a previous study was analyzed (Figure 1A). RNA sequencing followed by *de novo* assembly was performed to annotate transcripts expressed in the *A. bruennichi* venom gland. Sequences showing homology with known toxin peptides in the Uniprot database were identified. As a result, TBIU001927 showed high similarity to the U1-lycotoxinLs1k (E-value of 7e-24, 53% identity) and U29-aranetoxin-Av1a (E-value of 5e-55, 76% identity) sequences (Figure 1B). In addition, TBIU029815 was found to have significant homology to the U4-aranetoxinAv1a (E-value of 9e-05, 37% identity) and U4-aranetoxinAv1G sequences (Figure 1C). Sequences that showed homology were spider-derived peptides that were annotated for their toxin activity. Finally, mature 64-mer and 33-mer peptide sequences were identified from TBIU001927 and TBIU029815, respectively, by predicting signal peptides or propeptide regions from precursors.

**Figure 2:**
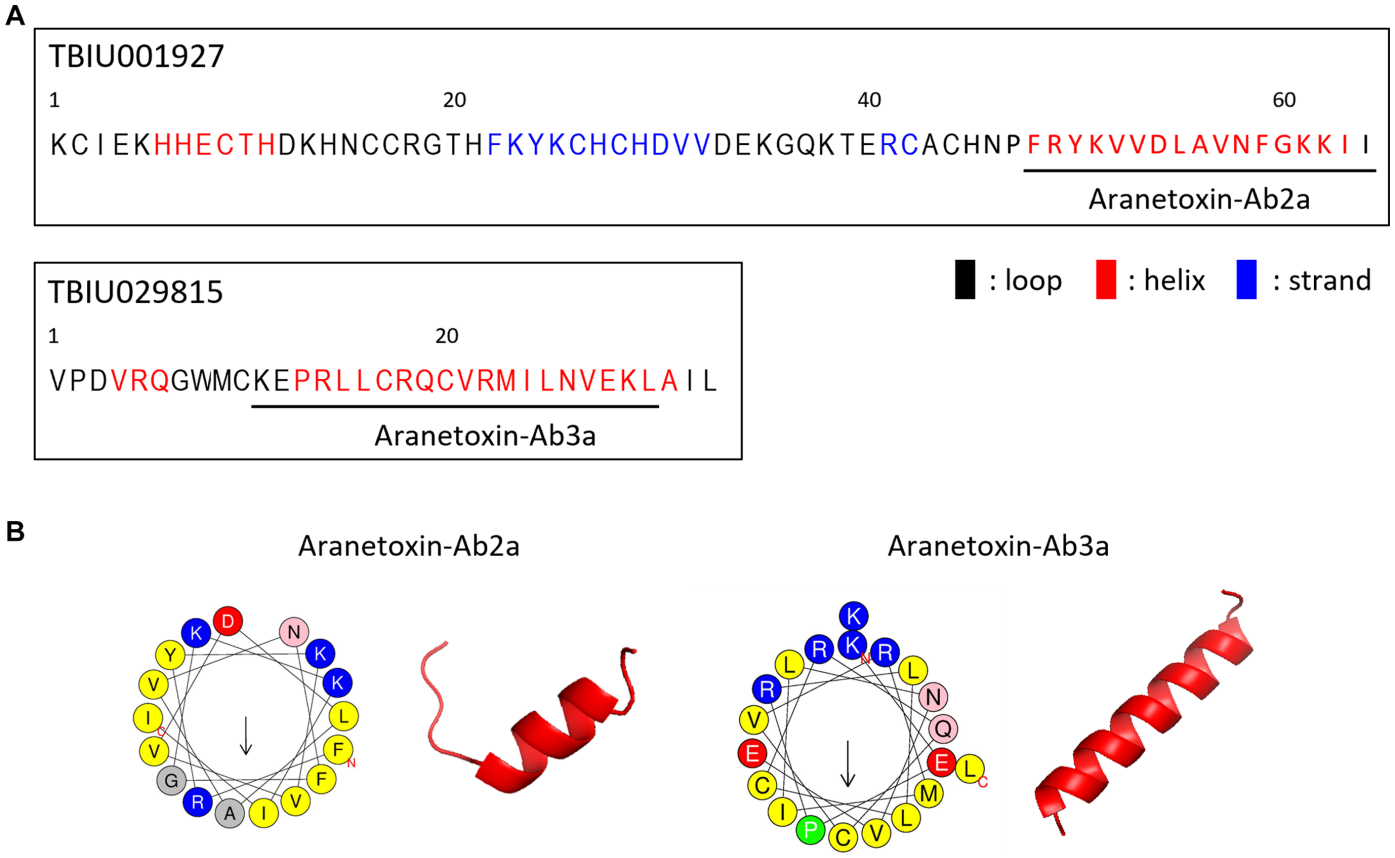
Structure of AATX-Ab2a and AATX-Ab3a. **(A)** Secondary structure predicted from TBIU01927 and TBIU029815 sequences is shown in black (loop), red (helix), and blue (strand). Potent AMP regions encoded in the original transcripts were named Aranetoxin-Ab2a (AATX-Ab2a) and Aranetoxin-Ab3a (AATX-Ab3a) and are shown underlined. **(B)** Amino acid configuration analysis and structural modeling of AATX-Ab2a and AATX-Ab3a are shown.

AMPs are the main components of animal venom and function by protecting the host from infection and aiding in hunting prey. Because TBIU01927 and TBIU029815 were identified as putative toxin peptides expressed in the *A. bruennichi* venom gland, further *in silico* analyses were conducted. Secondary structure analysis showed that the C-terminals of both transcripts had an α-helical structure. Accordingly, the segment from each transcript with high sequence homology was secured from the α-helix regions and was named Aranetoxin-Ab2a (AATX-Ab2a) and Aranetoxin-Ab3a (AATX-Ab3a) (Figure 2A). Amino acid composition and physiochemical analysis were subsequently performed. It was calculated that both peptides had a net charge above +2.9 and had helices with a hydrophobic face (Figure 2B). Moreover, the peptides were predicted as AMPs when analyzed with machine learning-based prediction tools (Table 1). As AATX-Ab2a and AATX-Ab3a showed general characteristics of AMP, the synthesis of these peptides was carried out for functional validation.

**Table 1:** Antimicrobial peptide prediction using machine learning-based prediction tools.

Tool	ADAM (SVM)	AmpGram	CAMP3 (RF)	DBAASP
Aranetoxin-Ab2a	AMP (2.33)	0.9482	AMP (0.9045)	AMP
Aranetoxin-Ab3a	AMP (0.52)	0.5552	AMP (0.5225)	AMP

### 3.2. Antimicrobial effects of AATX-Ab2a and AATX-Ab3a on pathogenic strains

**Figure 3:**
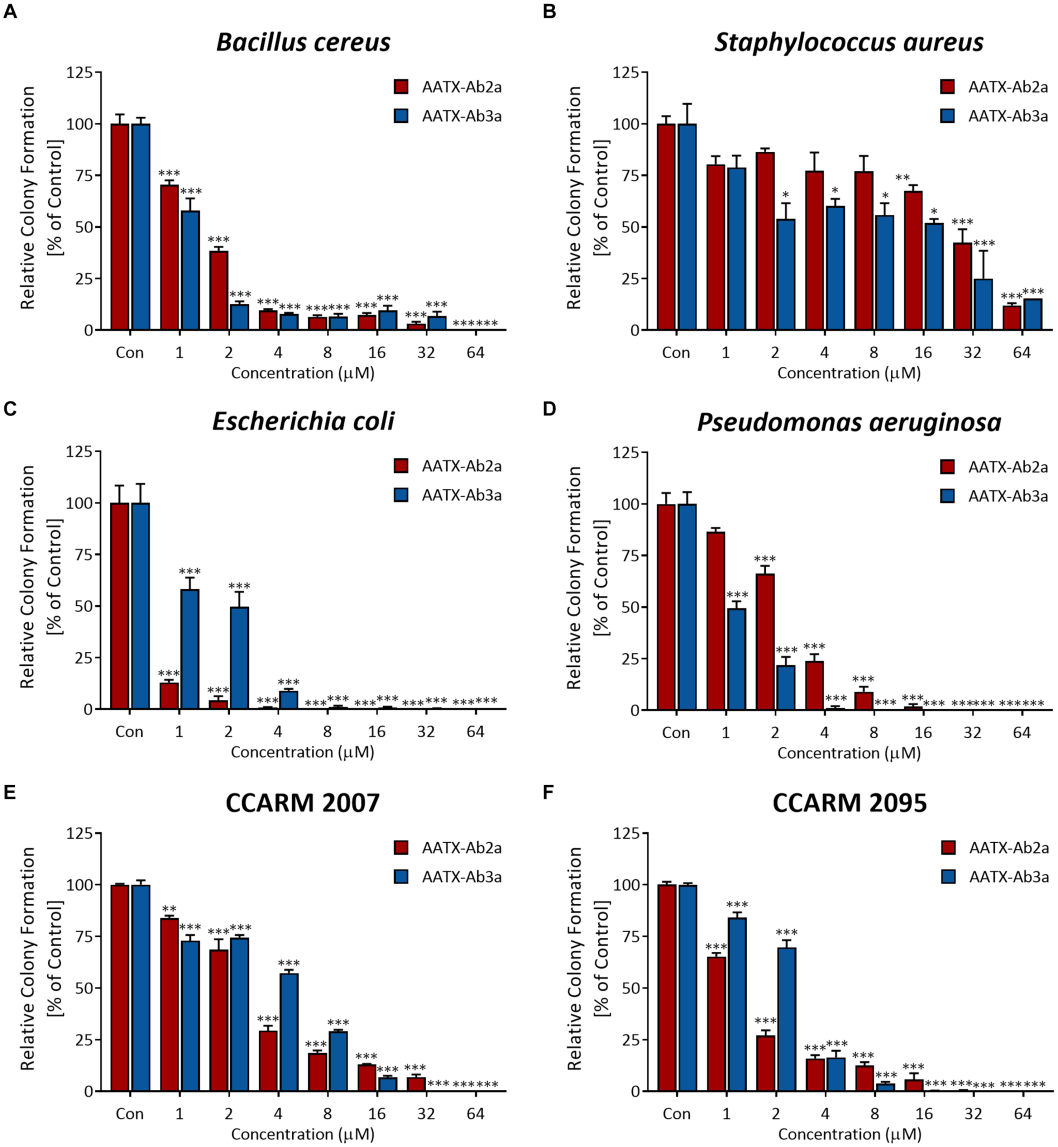
Antibacterial activity of AATX-Ab2a and AATX-Ab3a on pathogenic bacteria. **(A)** *Escherichia coli*, **(B)** *Pseudomonas aeruginosa*, **(C)** *Bacillus cereus*, **(D)** *Staphylococcus aureus*, **(E)** CCARM 2007, and **(F)** CCARM 2095 were subjected to CFU assays, and the antibacterial effects of AATX-Ab2a and AATX-Ab3a were determined. The results of triplicate experiments are presented as mean ± SEM. *p < 0. 05, **p < 0.01, ***p < 0.001 indicate significantly different activity com-pared with the control.

To confirm the antimicrobial activity of AATX-Ab2a and AATX-Ab3a, colony-forming assays were performed. Common pathogenic bacterial and fungal strains were treated with peptides ranging in concentration from 1 to 64 μM. The peptides inhibited the bacterial growth of Gram-positive strains, *B. cereus* and *S. aureus*, in a dose-dependent manner (Figures 3A,B). The colony formation of Gram-negative strains, *E. coli* and *P. aeruginosa*, also showed a significant decrease after AATX-Ab2a and AATX-Ab3a treatment (Figures 3C,D). Because the peptides exerted strong antibacterial effects on Gram-negative bacteria, MDR *P. aeruginosa* (MDR-PA) isolates were also tested against peptides. The CCARM 2007 and CCARM 2095 isolates, which are resistant to multiple antibiotics of the cephalosporin, ureidopenicillin, and quinolone groups, were used in this study. The results showed that MDR-PA CCARM 2007 and CCARM 2095 were susceptible to AATX-Ab2a and AATX-Ab3a, with a complete inhibition being observed at 64 μM (Figures 3E,F).

**Figure 4:**
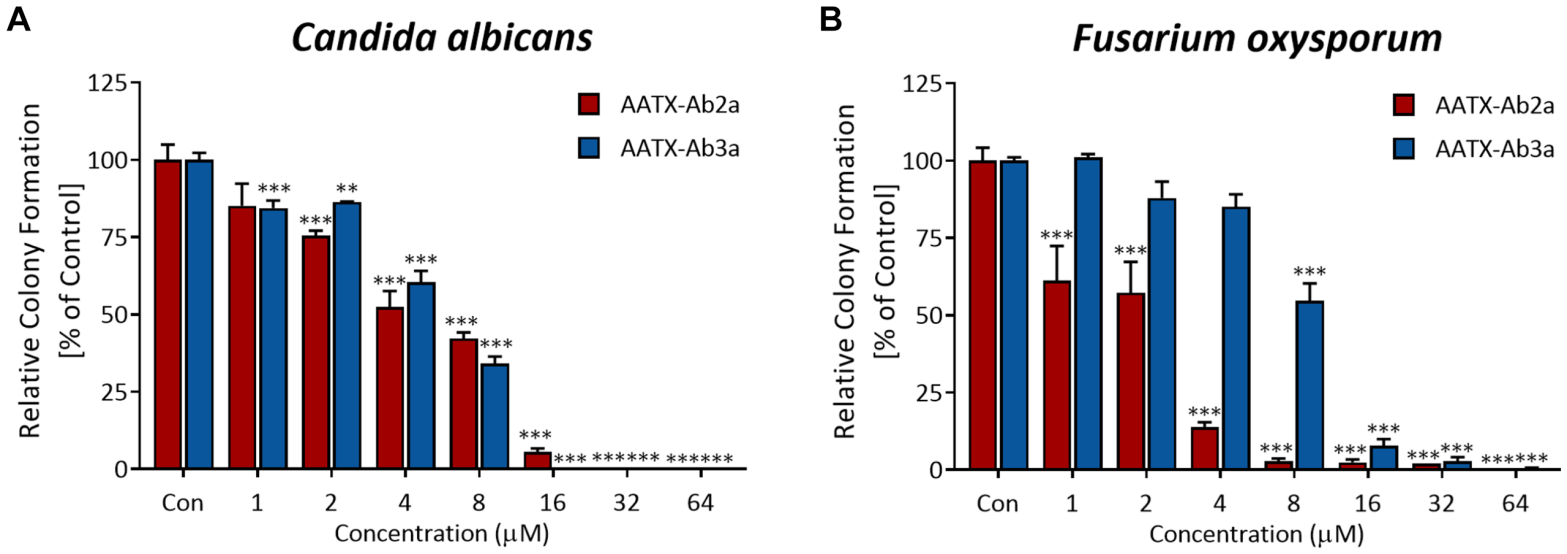
Antifungal activity of AATX-Ab2a and AATX-Ab3a on animal and plant fungi. **(A)** *Candida albicans* and **(B)** *Fusarium oxysporum* were susceptible to AATX-Ab2a and AATX-Ab3a, where peptides showed significant inhibition of growth. The results of triplicate experiments are presented as mean ± SEM. **p < 0.01, ***p < 0.001 indicate significantly different activity com-pared with the control.

Next, the anti-fungal effects of the peptides were tested on yeast *C. albicans* and filamentous fungus *F*. *oxysporum*, which are representative animal and plant pathogens, respectively. The growth of the two species was significantly inhibited by peptide treatment, showing 90% inhibition of growth when treated with 16 μM or higher concentration of peptides (Figures 4A,B). The results demonstrated the strong inhibitory effects of AATX-Ab2a and AATX-Ab3a on a broad range of pathogens, including Gram-positive, Gram-negative, MDR-PA, and fungal species. Accordingly, the mechanism of action of the peptides was further investigated.

### 3.3. Effects of AATX-Ab2a and -Ab3a on microbial membrane permeability

**Figure 5:**
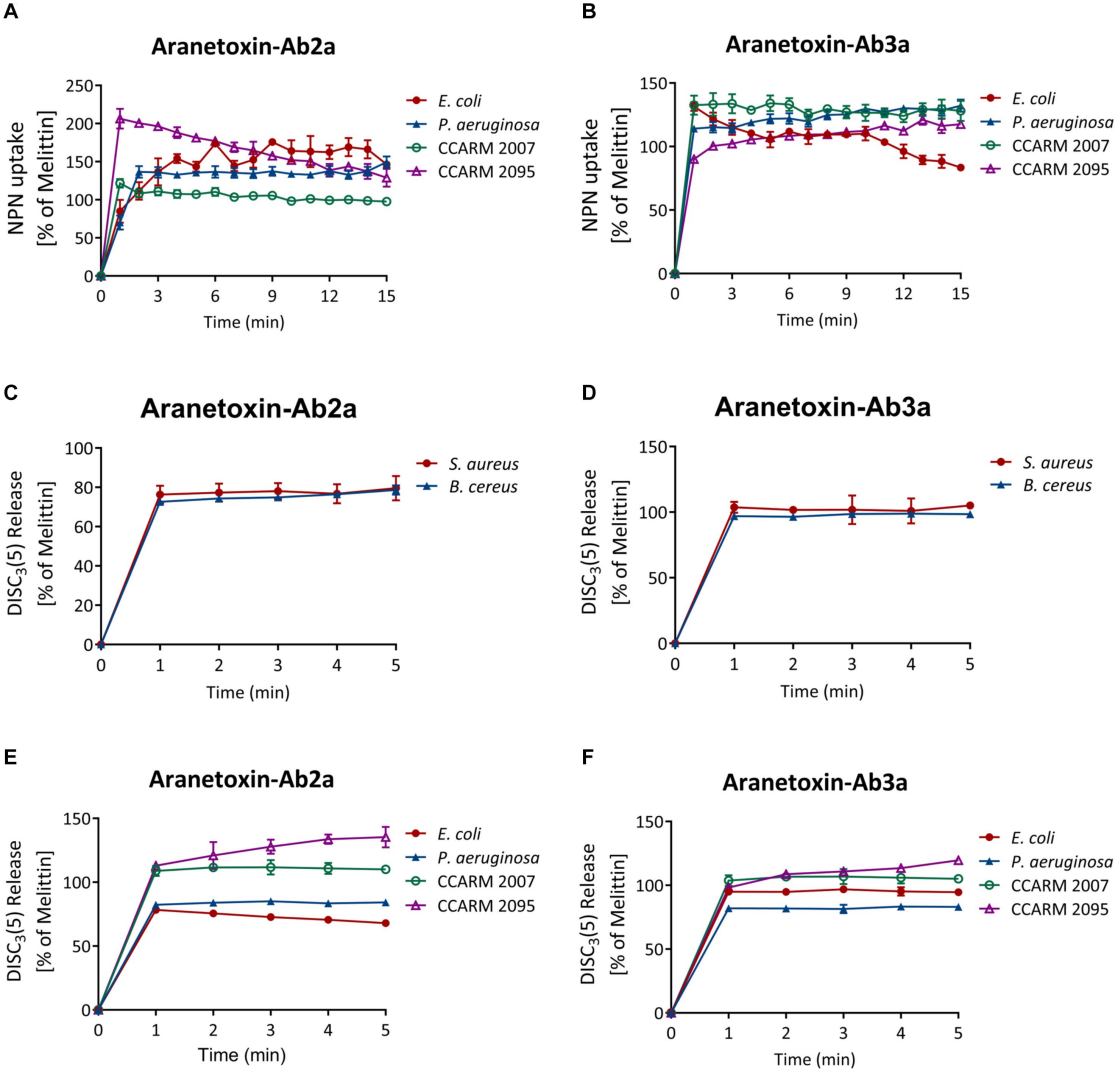
Effects of AATX-Ab2a and AATX-Ab3a on bacterial membranes. **(A,B)** The outer membrane disruption of Gram-negative strains was observed after peptide treatment with NPN uptake assays. The cytoplasmic membrane polarization of **(C,D)** Gram-positive and **(E,F)** Gram-negative bacteria was assessed using the DiSC_3_(5) fluorescent dye. The effects of AATX-Ab2a and AATX-Ab3a are presented relative to those of melittin.

NPN uptake assays were performed to measure the permeabilization of the outer membrane of Gram-negative bacterial strains. NPN emits strong fluorescent signals in hydrophobic conditions, as is the case when entering the inner portion of the membrane by pore formation. Fluorescence was recorded for 15 min for every 1 min after treating 16 μM of peptides (Figures 5A,B). The results showed a significant increase in fluorescence signal in both *E. coli* and *P. aeruginosa*. AATX-Ab2a and AATX-Ab3a induced rapid outer membrane disruption on MDR-PA as well, which was similar to or higher than that of 16 μM melittin treatment.

The fluorescent dye DiSC_3_(5) was used to assess the disruption of the bacterial cytosolic membrane. This dye fluoresces when leaked out of cells by membrane depolarization. DiSC_3_(5) was stabilized in the cytoplasm before the addition of the peptides. Likewise, treatment with 16 μM AATX-Ab2a and AATX-Ab3a induced an instant spike of signal in all of the bacterial cells (Figures 5C–F). Gram-negative bacteria were more susceptible to the peptides as the fluorescence intensity was similar to that of melittin treatment. Specifically, CCARM 2007 and CCARM 2095 were shown to be more susceptible to peptide treatment than *E. coli* and *P. aeruginosa*, showing higher fluorescence intensity. The overall results indicated that the two peptides are potent AMPs that can increase the permeability of both the outer and cytosolic membranes of bacteria.

**Figure 6:**
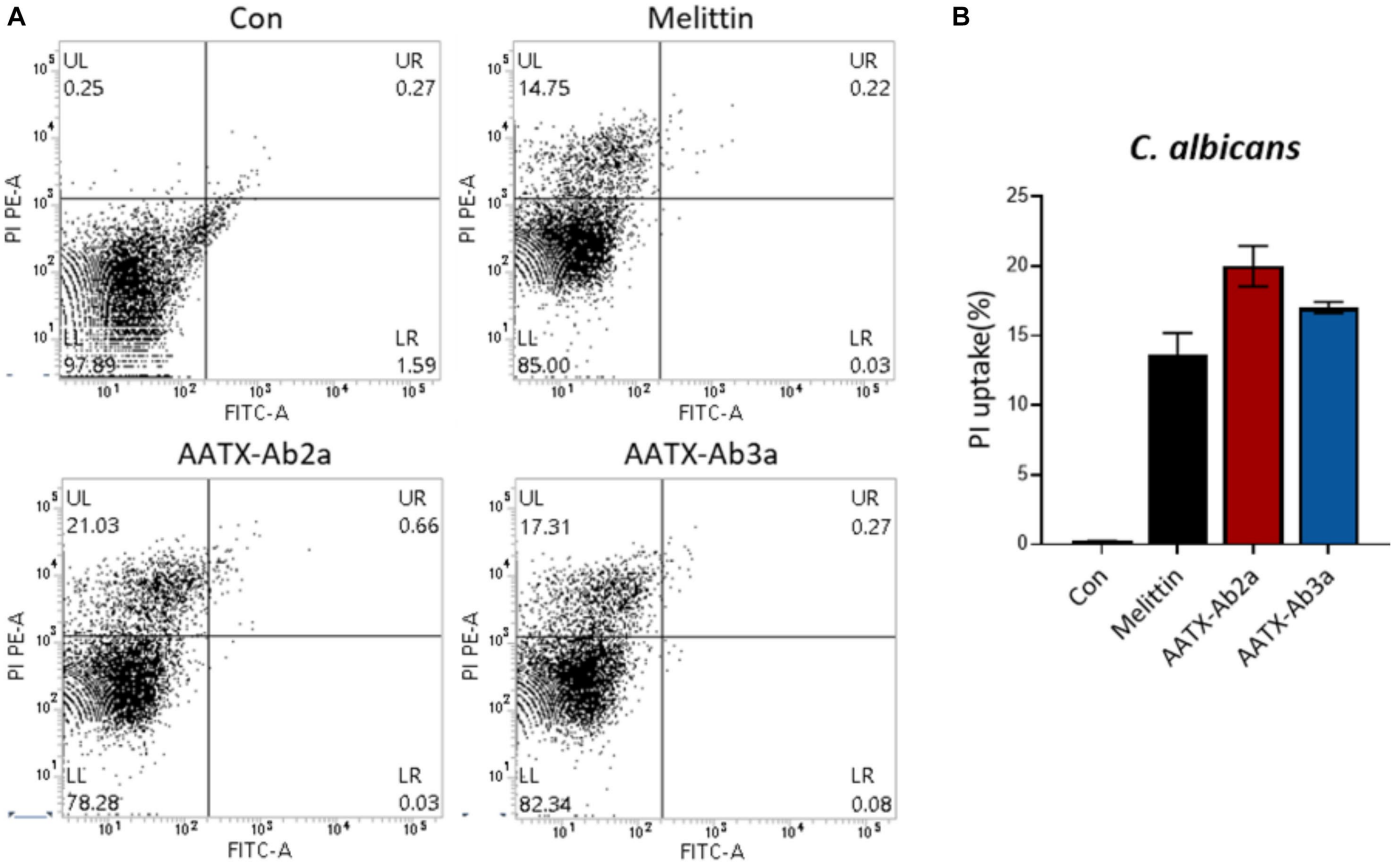
Cellular uptake of PI in *C. albicans* after AATX-Ab2a and AATX-Ab3a treatments. **(A)** Flow cytometry analysis was performed to determine the effects of peptides on fungal membrane integrity. PI-positive *C. albicans* cells of control and melittin-, AATX-Ab2a-, and AATX-Ab3a-treated groups are presented, and **(B)** the overall result is summarized.

**Figure 7:**
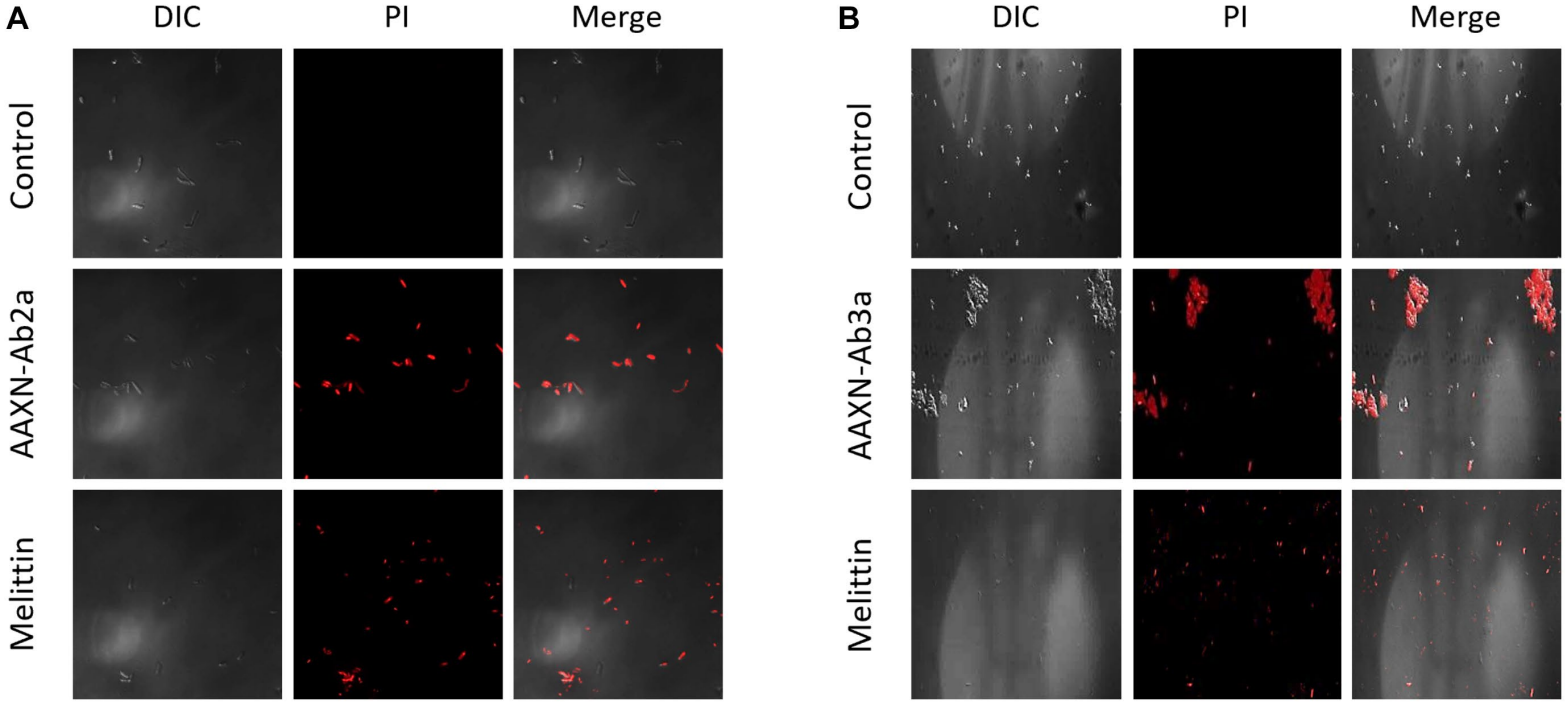
PI staining of *F*. *oxysporum* after AATX-Ab2 and AATX-Ab3a treatments. PI staining of *F*. *oxysporum* was visualized by confocal microscopy after incubating with **(A)** AATX-Ab2a or **(B)** AATX-Ab3a for 2 h. Melittin treatment in the same conditions was used as a positive control.

The effects of the peptides on fungal cell integrity were determined using propidium iodide (PI). PI is a red fluorescent dye that binds to nucleic acids and enters cells only when the plasma membrane is damaged. *C. albicans* was incubated with either PBS or 16 μM of AATX-Ab2a, AATX-AB3a, or melittin for 2 h, followed by flow cytometry analysis (Figure 6). Although almost every cell in the control group showed an intact membrane, 14.75% of *C. albicans* cells were stained with PI when treated with melittin. AATX-Ab2a and AATX-Ab3a treatments increased the proportion of PI-positive cells to 21.03 and 17.31%, respectively, indicating that the peptides induced a stronger membrane disruption than that of melittin. The effects of peptides on *F*. *oxysporum* were observed with confocal microscopy. After incubation with the peptides, red fluorescence was observed in both AATX-Ab2a- and AATX-Ab3a-treated groups, which was comparable to that of the melittin-treated group (Figures 7A,B). The overall results indicated that AATX-Ab2a and AATX-Ab3a interact with both bacterial and fungal membranes, increasing membrane permeability and causing cell damage.

### 3.4. AATX-Ab2a and AATX-Ab3a exhibit low cell toxicity

**Figure 8:**
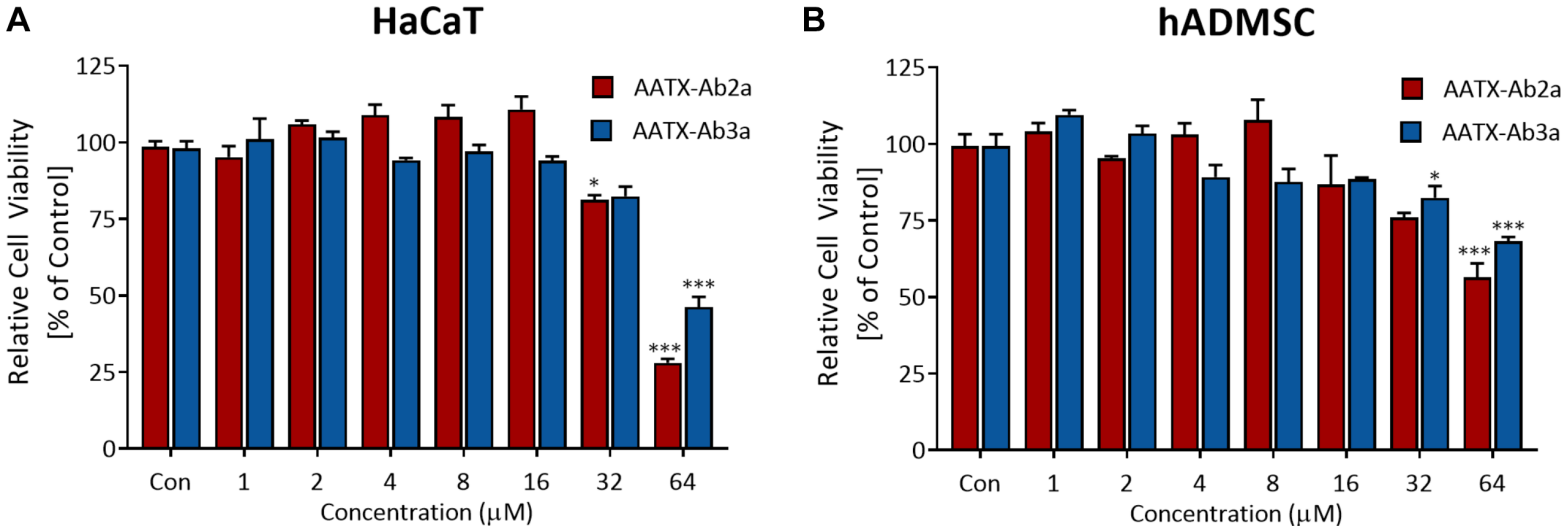
Assessment of cell viability upon AATX-Ab2 and AATX-Ab3a treatments. **(A)** HaCaT and **(B)** hADMSC were incubated with peptides at concentrations ranging from 1 to 64 μM for 24 h. Cell viability is expressed as relative to the control group. Results of triplicate experiments are presented as mean ± SEM. *p < 0.05, ***p < 0.001 indicate significantly different activity compared with the control.

In order to be used as antibiotic agents, candidate molecules should have low toxicity toward human cells and tissue. Thus, cell viability assays were performed to determine whether AATX-Ab2a and AATX-Ab3a are toxic to human cells. Immortalized human epithelial keratinocytes and human primary mesenchymal stem cells were used in this study. The cells were treated with AATX-Ab2a and AATX-Ab3a for 24 h, and cell viability was measured using WST-8. In both cell lines, no significant cytotoxicity was observed up to a concentration of 16 μM, and cell viability was maintained above 80% at 32 μM (Figure 8). AATX-Ab2a showed a stronger effect on cell viability than AATX-Ab3a at the highest concentration of 64 μM. It was demonstrated that AATX-Ab2a and AATX-Ab3a exhibited low cyto-toxicity to normal cells at concentrations with effective antibacterial and antifungal activities.

## 4. Discussion

Antibiotics have greatly contributed to human health since their discovery by saving countless lives from infections and related diseases. However, the use of conventional antibiotics is gradually being limited by the emergence of antibiotic-resistant strains (Wang et al., 2020; Chang et al., 2022; Larsson and Flach, 2022). Efforts to mitigate the antibiotic resistance crisis include restricting the use of antibiotics, controlling infections, and accelerating the diagnosis of the infectious agent (Aslam et al., 2021). However, discovering new antibiotics and broadening the spectrum of clinically usable substances is the fundamental solution, which highlights the importance of searching for new antimicrobial agents (Kalelkar et al., 2022). In this study, two novel AMPs with a wide range of antimicrobial activities were identified from venom gland transcripts of the *A. bruennichi* spider using an *in silico* approach.

Given the diversity of venom components and their range of functions, animal venom can be an excellent source of bioactive molecules (Yacoub et al., 2020). Specifically, spiders are known to have the highest diversity of peptides among venomous organisms. Indeed, spider venom-derived peptides with antibacterial, analgesic, antimalarial, and anti-arrhythmic activities have been reported (Saez et al., 2010). To discover antibacterial peptides, homology analysis was performed using an *A. bruennichi* venom gland transcript library, from which TBIU01927 and TBIU029815, which showed high levels of homology to spider toxin peptides, were identified. The mature peptide regions, which are secreted into the venom and have a specific function, were identified from the putative transcripts. *In silico* characterization and prediction were employed to secure a peptide region with high antimicrobial activity. As AMP is known to have an amphiphilic α-helical structure with a high positive net charge, the mature peptide was truncated at a site reflecting the general characteristics of AMPs. Finally, AI-based functional prediction was considered for selecting AATX-Ab2a and AATX-Ab3a.

Experimental validation revealed that AATX-Ab2a and AATX-Ab3a had broad spectrum of antimicrobial activity. Significant inhibition was observed against Gram-positive and Gram-negative pathogens, and complete inhibition was confirmed for MDR-PA isolates within the tested concentration range of the peptides. AMPs inhibit bacterial growth and induce cell death through pore formation while interacting rapidly and specifically with bacterial biomembranes (Melo et al., 2009). Using NPN and DiSC_3_(5) fluorescent dyes, it was confirmed that AATX-Ab2a and AATX-Ab3a also increased the permeability of the bacterial outer membrane and induced depolarization of the cytoplasmic membrane. These characteristics are advantageous for developing antibiotics, preventing drug resistance, and enabling efficient pathogen removal.

It is also important to search for new antifungal agents because there are fewer available antifungal drugs (Perfect, 2017). Fungal infections are also becoming a major problem in human health as well as in the food and agricultural industries (Fisher et al., 2020). Because fungi are eukaryotic organisms, it is important to find antifungal agents with specificity as they often affect animal cells as well (Mazu et al., 2016). AATX-Ab2a and AATX-Ab3a were confirmed to have significant suppression against both animal and plant fungal strains. In addition, the two peptides showed specificity for bacteria and fungi, as they exhibited low cytotoxicity toward the normal human cell lines HaCaT and hADMSC. The mode of action of the peptides were revealed to be membrane disruption through interaction with the pathogen’s biomembranes. Thus, we conclude that the peptides specifically target the cell wall and membrane components. Moreover, AATX-Ab3a has a relatively lower net charge than AATX-Ab2a, suggesting that it may be advantageous for biocompatibility and cytocompatibility.

As peptides inherently possess molecular diversity and target specificity, they are suitable for development as new antibiotics (Yount et al., 2006). In particular, target and/or function-specific derivatives can be generated through *in silico* analyses utilizing transcriptomic and proteomic data from venomous organisms (Roly et al., 2014). When peptides are to be used as therapeutic agents, their instability and insusceptibility should be considered (Rastogi et al., 2019). These problems can be overcome by designing peptides with enhanced activity and biocompatibility through functional prediction and characterization aided by computational analysis (Cardoso et al., 2020). The workflow described in the present study allowed the successful identification of two novel AMPs derived from spider toxin that exhibited strong inhibitory activity against pathogens, including MDR-PA and fungi. This approach will aid the future search and development of bioactive substances by expanding the utilization of biological resources.

## Data availability statement

The original contributions presented in the study are included in the article/supplementary material, further inquiries can be directed to the corresponding author.

## Author contributions

MKS and I-WH conceptualized the study, performed the *in silico* analyzes and experiments, and wrote the manuscript. B-YJ and K-BB validated and analyzed the data. JSY coordinated the project and acquired the funding. J-SS supervised the study and edited the manuscript. All authors contributed to the article and approved the submitted version.

## Funding

This research was supported by the Basic Science Research Program through the National Research Foundation of Korea (NRF) funded by the Ministry of Education (NRF-2022R1A6A1A03053343) and grants from the National Institute of Biological Resources (NIBR), funded by the Ministry of Environment (MOE) of the Republic of Korea (NIBR202333202 and NIBR202304103).

## Conflict of interest

The authors declare that the research was conducted in the absence of any commercial or financial relationships that could be construed as a potential conflict of interest.

## Publisher’s note

All claims expressed in this article are solely those of the authors and do not necessarily represent those of their affiliated organizations, or those of the publisher, the editors and the reviewers. Any product that may be evaluated in this article, or claim that may be made by its manufacturer, is not guaranteed or endorsed by the publisher.

## Footnotes

^1^ https://pepcalc.com/protein-calculator.php

^2^ http://xtalpred.godziklab.org/XtalPred-cgi/xtal.pl

^3^ http://www.cbs.dtu.dk/services/SignalP

^4^ http://www.arachnoserver.org/spiderP.html

^5^ http://mobyle.rpbs.univ-paris-diderot.fr/cgi-bin/portal.py#forms::PEP-FOLD3

^6^ http://heliquest.ipmc.cnrs.fr/cgi-bin/ComputParams.py

^7^ http://bioinformatics.cs.ntou.edu.tw/ADAM

^8^ http://biongram.biotech.uni.wroc.pl/AmpGram/

^9^ http://www.camp3.bicnirrh.res.in

^10^ http://dbaasp.org/
